# Physical exercise and major depressive disorder in adults: systematic review and meta-analysis

**DOI:** 10.1038/s41598-023-39783-2

**Published:** 2023-08-14

**Authors:** Édison Andrés Pérez Bedoya, Luisa Fernanda Puerta-López, Daniel Alejandro López Galvis, Diego Alejandro Rojas Jaimes, Osvaldo Costa Moreira

**Affiliations:** 1 Department of Physical Education of the Federal University of Viçosa, Av. Peter Henry Rolfs, S/N - Campus Universitário, Viçosa, MG 36570-900 Brazil; 2 Department of Physical Education of Antioquia University, Medellín, Colombia; 3 Department of Physical Education of Llanos University, Villavicencio, Colombia

**Keywords:** Psychology, Human behaviour

## Abstract

The objective of this study was to assess the benefits and potential risks associated with different physical exercise modalities for managing symptoms in adults with major depressive disorder who were not receiving second-generation antidepressants or cognitive behavioral therapy. A systematic review and meta-analysis of randomized controlled trials (RCTs) were conducted. The search included multiple databases: Medline, Cochrane Central Register of Controlled Trials (CENTRAL), Embase, PsycInfo, Web of Science, Clinical Trials repository, gray literature, and manual search. No language restrictions were applied. Eligible studies involved RCTs of adults with major depressive disorder who were not on antidepressants or receiving psychological therapy, comparing various exercise modalities with second-generation antidepressants or cognitive behavioral therapy, body-mind exercise, or no exercise interventions. Nine RCTs involving 678 adults were analyzed. The pooled results indicated a small clinical effect favoring exercise in reducing depressive symptoms, although the difference was not statistically significant (SMD = 0.27, 95% CI [− 0.58, 0.04], P = 0.09). Subgroup analyses suggested that intervention duration, frequency, intensity, supervision, age, overweight/obesity status, and diagnosis of depression could influence treatment outcomes. A sensitivity analysis was conducted for studies with controls without exercise interventions and a low risk of bias in the domains related to the randomization process and deviations from the intended interventions. The results showed that there are no statistically significant differences when interventions are compared with medication and body-mind exercise (p = 0.12, I^2^ = 78%). Furthermore, the analysis showed a moderate effect size favoring exercise, but no statistically significant difference between groups (p = 0.05), with high heterogeneity (I^2^ = 85%). The evidence quality was generally low to very low, and methodological limitations compromised the certainty of the findings. Adverse events associated with exercise were manageable. The study emphasizes the need for well-designed RCTs to provide clearer insights into the potential benefits of exercise in managing major depressive disorder symptoms. Caution is warranted in interpreting these results due to the limitations of the included studies.

*Systematic review registration:* PROSPERO CRD42022356741.

## Introduction

Major depressive disorder (MDD) is indeed more prevalent in women than in men, with a prevalence rate of 14.4% in women and 11.5% in men^1^. It is characterized by persistent symptoms such as a depressed mood, loss of interest, and a reduced ability to experience pleasure in daily activities for a minimum duration of two weeks^1–5^. It typically emerges in early adulthood, with an average onset age of around 20–25 years^6^. The prevalence tends to be higher in developed countries among individuals aged 16 years and above^7,8^. In 2020, it was estimated that 264 million people worldwide were affected by MDD^3^. Individuals with this disorder are at an increased risk of developing various comorbidities, including diabetes mellitus, cardiovascular morbidity and mortality, lower back pain, and a decline in overall quality of life^9,10^. It has profound implications for both individual and public health. It is the leading cause of suicide deaths worldwide, with an estimated incidence of up to 800,000 suicides annually^11^. Additionally, it has emerged as an independent risk factor for all-cause mortality, further underscoring its impact on overall health^12^. Prior to the COVID-19 pandemic, it was already the second leading cause of disability globally, and since 2020, there has been a noticeable increase in its incidence, affecting approximately 53.2 million individuals^7,8^. The economic burden associated with it is substantial; in the United States, the economic losses were around $210.5 billion in 2010, and by 2020, they had escalated to nearly $390 billion per year^1^. Interestingly, effective treatment could potentially yield a net global economic benefit of $230 billion by 2030^13^. Despite the high burden and economic impact, mental health expenditures receive only a small fraction of government health budgets, with approximately 2% allocated to mental health worldwide, as reported by UNICEF^7^.

Clinical practice guidelines recommend the use of psychotherapy and/or pharmacotherapy for MDD treatment^14^. However, these approaches may face barriers to adherence: stigma surrounding mental health and concerns about medication-related adverse effects (such as constipation, diarrhea, dizziness, headache, insomnia, nausea, decreased sexual desire, and somnolence) can significantly impact treatment acceptance and adherence^14,15^. Therefore, there is an urgent need to explore non-pharmacological and patient-centered strategies that are safe, feasible, and easily integrated into the daily routines of adults.

Physical exercise (PE) interventions have been shown to effectively alleviate depressive symptoms in adults and are recommended by international guidelines, including the Canadian Network for Mood and Anxiety Treatments and the American College of Physicians^14–16^.

The evaluation of non-pharmacological therapies, including exercise, has been the focus of various guidelines, systematic reviews, and meta-analyses^2–4,12,14–17^. However, it is worth noting that not all these reports have specifically considered PE in their recommendations. Some guidelines have primarily focused on cognitive-behavioral therapy or second-generation pharmacological therapy as the primary treatment options, potentially overlooking the potential benefits of exercise^14^. These guidelines strongly recommend these therapies with moderate certainty.

However, there is evidence suggesting that certain forms of exercise could serve as monotherapy for individuals with mild to moderate MDD or as adjunctive treatment for those in the moderate to severe stages of the disorder^15^. It is important to acknowledge that the effect of exercise on symptomatology may vary from moderate to small, and some studies included in these reports have a high risk of bias^15^.

For example, a Cochrane review and a systematic review included a diverse population, encompassing individuals with a range of characteristics, including some who were receiving drug therapy in combination with exercise interventions. These reviews also included healthy individuals who exhibited depressive symptoms, in addition to those specifically diagnosed with MDD^4,18^. Similarly, network meta-analyses have incorporated older adults with dementia, some of whom were prescribed antidepressant medication, in their analyses^17^.

It is worth noting that while systematic reviews have reported a moderate and mild effect of exercise on this disorder, there remains uncertainty regarding the optimal type, intensity, duration, and frequency of exercise that may be most effective^2,12,16^. The lack of evaluation of the strength and certainty of results in previous systematic reviews and meta-analyses is indeed a significant concern. Only the Cochrane review and network meta-analysis assessed the level of certainty in the results, and the network meta-analysis found varying levels of certainty for different exercise interventions^4,17^. This highlights the need for a comprehensive evaluation of the evidence using robust methodologies.

It is essential to determine the level of confidence we can have in the potential mild to moderate effect of exercise. None of the reviews published in the last five years have assessed the strength and certainty of the results using the GRADE approach, which is a rigorous framework for evaluating the quality of evidence^19,20^. As a result, our understanding of the harms associated with PE practice for this population is limited since previous reviews have not adequately assessed adverse events.

Therefore, the aim of this meta-analysis is to investigate the effect of physical exercise in reducing depressive symptoms in adults diagnosed with MDD who are not receiving treatment. Additionally, we will assess the effect of exercise on quality of life and examine any adverse events associated with the interventions.

## Methods

### Protocol and registration

The study was registered in PROSPERO with the registration number CRD42022356741. The protocol adhered to the PRISMA (Preferred Reporting Items for Systematic Reviews and Meta-Analyses) protocol (PRISMA-P)^21^, and the final report was prepared in accordance with the recommendations of the PRISMA Statement^22^.

### Study eligibility criteria

Two reviewers, EAPB and LFPL, independently extracted and analyzed the references from Rayyan QCRI^23^. They conducted their analysis in a blinded manner and assessed the trials based on the predetermined eligibility criteria. Any discrepancies that arose between the reviewers were resolved by a third reviewer, DALG.

The PICOTS acronym was used to define the inclusion criteria for this systematic review and meta-analysis^24^. The following criteria were applied.

#### Participants

Adults of both sexes aged 18 years or older diagnosed with MDD according to the DSM-5™ Diagnostic Criteria reference guide^25^. Participants should not have been using antidepressant medication or undergoing psychological therapy prior to the exercise interventions. They may or may not have had chronic communicable or non-communicable diseases.

#### Interventions

Randomized controlled trials (RCTs) examining different modalities of PE, including aerobic training (AT), resistance exercise (RE), combined exercise (CE), and multicomponent exercise (MCE).

#### Comparators or control conditions

The interventions were compared to treatment recommended by American College of Physicians^14^ (second-generation antidepressants or cognitive behavioral therapy), body-mind exercise (Yoga, Tai chi, Qi gong, stretching exercise), and no exercise interventions.

#### Outcomes

The primary outcome of interest was depressive symptoms^26–28^. Secondary outcomes included adverse events or damage (such as dizziness, headache, blurred vision, and chronic muscle pain), quality of life, and mortality.

Studies were excluded from the meta-analysis if they met any of the following criteria:

1. RCTs in progress or those that conducted PE interventions with pregnant or breastfeeding women.
2. RCTs with comparators involving nutritional proposals.
3. Studies that included adults with clinical diagnosis of anxiety or bipolar disorder.
4. Studies with a population experiencing posttraumatic stress disorder.
5. Studies that did not provide sufficient information on the components of frequency, intensity, time, volume, and progression (FITVP).

The quality of the included RCTs in this systematic review and meta-analysis was assessed using the Study Quality Assessment Tool and Exercise Reporting Tool (TESTEX)^29^. The TESTEX scale is a quality assessment tool specifically designed for exercise training studies. It focuses on evaluating the quality and reporting of exercise training trials, with a particular emphasis on criteria relevant to exercise specialists. The scale includes criteria that may not be mentioned in other quality assessment tools, such as the transition from a sedentary control group to an exercise group, the periodic adjustment of exercise training intensity based on physical training adaptation, and the detailed reporting of exercise program characteristics. By using the TESTEX scale, researchers and exercise specialists can assess the quality and reporting of exercise training studies in a comprehensive and specific manner.

The reviewers, LFPL and DALG, independently assessed the quality of the studies based on predetermined criteria. Only RCTs with high methodological quality, scoring between 12 and 15 points on the TESTEX, were included in the metanalysis.

### Search procedures and study inclusion

The systematic search for eligible studies was conducted independently and in a blinded manner by two reviewers, EAPB and LFPL. The search included international electronic databases such as Medline (via Ovid), Cochrane Central Register of Controlled Trials (CENTRAL), Embase, PsycInfo, Web of Science, and the Clinical Trials repository (clinicaltrials.gov). Additionally, gray literature repositories including OpenSIGLE, PsycEXTRA, Healthcare Management Information Consortium (HMIC), and the National Technical Information Service (NTIS) were searched following the guidelines outlined in the Cochrane Handbook^30^.

To ensure thoroughness, a manual search of the reference lists of selected articles, previous systematic reviews, and meta-analytic studies was conducted to identify any potentially relevant studies that may have been missed in the electronic searches.

Any discrepancies or disagreements regarding the inclusion of a study were resolved through consensus discussions involving a third author, DARJ. There were no language or publication year restrictions, allowing for a comprehensive range of studies to be considered.

The specific search terms used in the systematic search can be found in Supplement 1 of the review, providing transparency, and allowing for replication of the search strategy.

Two authors, EAPB and LFPL, performed the study selection and data extraction, as well as assessed the risk of bias among the included studies. Disagreements between the two authors were resolved by another author, OCM. Additionally, two independent reviewers, EAPB and DALG, who were blinded to each other's assessments, utilized the Consensus on Exercise Reporting Template (CERT)^31^ to evaluate the included RCTs.

The risk of bias assessment was performed independently and in a blinded manner by the review group consisting of EAPB and LFPL. The Cochrane Revised Risk of Bias Tool for Randomized Trials (RoB2)^32^ was employed for this assessment. The severity of adverse events was assessed and graded using version 5.0 of the "Common Terminology Criteria for Adverse Events" (CTCAEv5.0)^33^. Adverse events were categorized into different grades based on their severity. Grade one adverse events may include muscle events, chest pain, and muscle/joint pain. Grade two adverse events may involve mood disturbances such as worsening of MDD or antidepressant-related follow-up. Grade three adverse events may encompass unspecified medical reasons, medication-related adverse events such as dizziness, drowsiness, agitation, or diarrhea, medical contraindications, new medical conditions, psychiatric emergencies related to mood disturbances, or admission to psychiatry. Grade four adverse events may refer to mood disturbances specifically related to suicidal ideation. Finally, grade five adverse events will be recorded in case of death by suicide.

To evaluate the certainty and strength of evidence in the findings provided by the included RCTs, the Grading of Recommendations, Assessment, Development and Evaluation (GRADE) approach^19,20^ was utilized. EAPB and LFPL, as independent and blinded reviewers, employed this approach to evaluate the quality of evidence and assign it to one of four levels: high certainty, moderate certainty, low certainty, or very low certainty. The Measurement Tool to Assess systematic Reviews (AMSTAR 2)^34^ was used to evaluate the quality of this review.

### Statistical analysis

For continuous outcomes, the study included group sizes, mean values, and standard deviations (SD) were compared. Pooled effects were calculated using an inverse variance model. Since some studies reported data from different instruments, the effects were evaluated based on the standardized mean differences (SMD) of PE interventions on the results obtained from questionnaires that measure symptoms related to MDD and the perception of quality of life. The corresponding 95% confidence intervals (CI) were established, with statistical significance set at p < 0.05. SMD was calculated to determine Cohen's d for each study, and Hedges' g was used to account for potential bias in small sample sizes. The interpretation of SMD followed Cohen's guidelines, where SMD values < 0.2 were considered trivial, 0.2–0.3 as small, 0.5 as moderate, and > 0.8 as large^35^.

The adverse events were analyzed as dichotomous outcomes, and a Mantel–Haenszel random effects model was used to pool and compare the total number of events in the AT, RE, CE, and MCE groups versus second-generation antidepressants or cognitive behavioral therapy, BME, or no exercise interventions. The risk difference (RD) was calculated with a 95% confidence interval (CI), and a positive value for RD indicated a favorable safety profile for PE. RD was chosen as the effect measure to ensure that RCTs reporting zero adverse events (indicating no difference between exercise and usual care) were not excluded from the meta-analysis. Statistical heterogeneity was assessed using the Higgins test (I^2^) and classified according to the Cochrane Manual: negligible heterogeneity (0% to 40%), moderate heterogeneity (30% to 60%), substantial heterogeneity (50% to 90%), and considerable heterogeneity (75% to 100%)^30^.

A random effects model^36^, was employed, assuming potential differences between the included studies and aiming to examine discrepancies among them. Subgroup analyses^37^ were conducted to explore the effects of PE, age, sex, health and training status, body composition, frequency, intensity, duration, and modes of PE. Additionally, a sensitivity analysis was performed for studies that had control conditions without PE interventions and demonstrated a low risk of bias in the domains related to the randomization process and deviations from the intended interventions. These two domains were selected based on their significance in assessing the quality of RCTs. The domain related to the randomization process evaluates whether the allocation sequence was randomized, adequately concealed, and if any initial differences between the intervention groups suggest a problem with the randomization process. Randomization helps to ensure that known and unknown prognostic factors, such as disease severity or comorbidities, are balanced between the intervention groups. This reduces the potential for bias in the assignment of individual participants to interventions. The most important elements assessed in RCTs for randomization include the generation of the allocation sequence (randomization elements) and the concealment of the allocation sequence (preventing participants or trial staff from knowing about upcoming assignments). The other domain selected for the sensitivity analysis is related to deviations from the intended interventions, which assesses performance bias. This domain considers whether there were any deviations from the trial protocol, such as administering additional interventions that are inconsistent with the protocol or not implementing the protocol interventions as intended. It also evaluates the participants' compliance with the assigned intervention. One way to minimize this bias is through blinding or masking, where the participants or trial staff are unaware of the assigned interventions. By conducting a sensitivity analysis focusing on these domains, we aimed to evaluate the robustness and reliability of the findings, ensuring that studies with high methodological quality and adherence to the intended interventions were given additional consideration^38^. Publication bias was not assessed due to the insufficient number of studies (less than 10) required for such analysis. All meta analyses were performed by two reviewers (EAPB and OCM) using RevMan 5.4^30^, and an author (LFPL) reviewed the extracted data for verification.

## Results

### Literature identification

**Figure 1:**
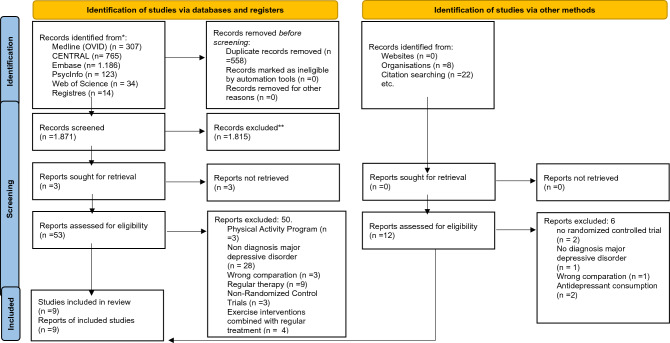
Preferred reporting items for systematic reviews and meta-analyses (PRISMA) flow chart of the study selection.

The initial search identified a total of 2429 studies. After removing duplicates, 558 studies were left. Following the screening of titles and abstracts, 1871 studies were excluded. Subsequently, 53 RCTs were assessed in full text by reviewers EAPB and LFPL. Among them, 50 trials did not meet the PICOTS criteria outlined in this review, and the reasons for their exclusion are provided in Supplement 1. After applying the TESTEX tool^29^, two studies^39,40^ were excluded from the quantitative synthesis (Supplement 1). Finally, nine trials were included for qualitative synthesis. A visual representation of the search results can be found in the PRISMA flow chart in Fig. 1*.*

### Demographic and study characteristics

The review included a total of nine RCTs with a combined sample size of 678 adults. Of these, 211 participants (31.12%) were men and 467 (68.88%) were women. The age range of the participants varied from 20 to 72 years old. The earliest publication included in the review was from 1996, and the most recent was from 2016^39–47^. It is worth noting that Blumenthal^43^ and Herman^42^ used the same sample in their studies. Most of the research was conducted in the United States of America, with six trials (66.66%) taking place in this country^42–47^. Denmark contributed two studies (22.22%)^39,41^, and Iran contributed one study (11.11%)^40^. In terms of body composition, some studies reported that the participants were overweight (three studies, 33.33%)^39,41,47^, obese (one study, 11.11%)^45^, or a combination of overweight and obesity (one trial, 11.11%)^46^. Four RCTs (44.44%) did not provide detailed information about the physical characteristics of the participants^40,42–44^.

In two trials (22.22%), participants with endocrine, cardiac, pulmonary, and orthopedic disorders were included^42,43^. Additionally, one RCTs (11.11%) included adults diagnosed with chronic noncommunicable diseases^45^. In several studies, the intervention was conducted with sedentary individuals (five studies, 55.55%)^39,41,45–47^. Furthermore, one study (11.11%) reported a population without employment^41^, while three RCTs (33.33%) included both unemployed and full-time workers^42–44^. The assessment of MDD symptomatology was conducted using the Hamilton Depression Scale (HAM-D) in four studies (44.44%)^39,41,45,46^, the Beck Depression Inventory (BDI) in four studies (44.44%)^40,42,43,47^, and both HAM-D and BDI scales in one study (11.11%)^44^. For more details on the characteristics of the participants, please refer to Table 1*.*

**Table 1:** Characteristics of the studies included.

Study, year, country	Characteristics of the participants									Depressive symptoms instrument	Results of the interventions std. mean difference, 95% CI	TESTEX score
	Age (years)	Participants, n	Male, n	Female, n	Sex (%)	Body composition	Health condition	Physical exercise and physical activity status	Occupational status (%)			
Krogh, 2012 Denmark	INT: 39.7 ± 11.3 CON: 43.4 ± 11.2	115 INT: 56 CON: 59	INT: 16 CON: 22	INT: 40 CON: 37	Men (33) and women (67)	Overweight	No chronic disease diagnosis	Sedentary behavior	Unemployed INT: 35.7 CON: 45.7	HAM-D	At the end of the interventions, no significant differences were found between the groups in terms of depressive symptoms 0.12, [− 0.24, 0.49]	14
Krogh, 2014 Denmark	INT:39.8 ± 11.7 CON: 43.8 ± 112.2	79INT: 41CON: 38	INT: 11 CON: 15	INT: 30CON: 23	Men (32.9) and women (67.1)	Overweight	No chronic disease diagnosis	Sedentary behavior	Not reported	HAM-D	They found an association between an increase in hippocampal volume, improved depression, and verbal memory independent of exercise Not included in meta-analysis	11
Singh, 1996 USA	INT: 70.0 ± 1.6 CON: 72.0 ± 1.9	28 INT: 15 CON: 13	INT: 5 CON: 6	INT: 10 CON: 7	Men (39.29) and women (60.71)	Overweight	No chronic disease diagnosis	Sedentary behavior	Not reported	BDI	Progressive resistance exercise significantly reduced all measures of depression compared to control − 0.43, [− 1.18, 0.32]	12
Herman, 2002 USA	INT:57 ± 5.8 CON: 57 ± 7.0	101 INT: 53 CON: 48	INT: 14 CON: 10	INT: 39 CON: 43	Men (23.76) and women (76.23)	Not reported	Endocrine, Cardiac, Pulmonary, Orthopedic disorders	Not reported	Unemployed35employed65	BDI	No treatment group differences in remission rate were found − 0.26, [− 0.65, 0.13]	13
Blumenthal, 1999 USA	INT:57 ± 5.8 CON: 57 ± 7.0	101 INT: 53 CON: 48	INT: 14 CON: 10	INT: 39 CON: 38	Men (23.76) and women (76.23)	Not reported	Endocrine, Cardiac, Pulmonary, Orthopedic disorders	Not reported	Unemployed35employed65	BDI	After 16 weeks of treatment, there were no statistically significant differences between the groups in terms of scores on BDI 0.13, [− 0.26, 0.52]	12
Khatri, 2001 USA	56.73 ± 6.45	84 INT: 42 CON: 42	20	64	Men (23.80) and women (76.19)	Not reported	Not reported	Not reported	Unemployed32employed68	HAM-DBDI	Both groups demonstrated significant improvements in depressive symptoms as HAM-D and BDI 0.28, [− 0.15, 0.71]	12
Blumenthal, 2007 USA	INT:52 ± 7 INT: 53 ± 8 CON: 52 ± 8	153 INT:51 INT: 53 CON: 49	INT:12 INT: 14 CON: 12	INT:39 INT: 39 CON: 37	Men (24.83) and women (75.17)	Obesity	chronic disease diagnosis	Sedentary behavior	Not reported	HAM-D	All treatment groups had lower HAM-D scores after treatment; scores for the active treatment groups were not significantly different from the placebo group − 0.16, [− 0.55, 0.23]	13
Sadeghi, 2016 Iran	INT: 20.93± 1.06 CON: 21.12± 1.25 CON: 20.92 ± 1.20	46 INT: 16 CON: 16 CON: 14	INT: 13 CON: 12 CON: 11	INT: 3 CON: 4 CON: 3	Men (78.26) and women (21.73)	Not reported	Not reported	Not reported	Not reported	BDI-II	Aerobics, compared to the control group, causes more reductions in depression variable Not included in meta-analysis	6
Dunn, 2005USA	INT: 35.8 ± 6.1 INT: 37.7 ± 5.1 INT: 33.2 ± 6.7 INT: 37.9 ± 6.3 CON: 34.5 ± 7.3	72 INT: 16 INT: 15 INT: 17 INT: 15 CON: 9	INT: 3 INT: 4 INT: 4 INT: 3 CON: 4	INT: 13 INT: 11 INT: 13 INT: 12 CON: 5	Men (25) and women (75)	Overweight and Obesity	Not reported	Sedentary behavior	Not reported	HAM-D	The main effect of energy expenditure in reducing HAM-D scores at 12 weeks was significant − 2.74, [− 3.92, − 1.55]	15

*HAM-D* Hamilton Depression Rating Scale, *HDRS* The Hamilton Rating Scale of Depression, *BDII* Beck Depression Inventory, *BDI-II* Beck Depression Inventory-ll, *INT* Intervention, *CON* Control, *Std. Mean Difference* Effect size, *CI* Confidence interval.

### Description of physical exercise interventions

The exercise modality that was most studied was AT, which was examined in eight RCTs (88.88%)^39–46^. Only one RCTs (11.11%) focused on RE^47^. All PE interventions were supervised^39–47^. In one study (Blumenthal, 2007), in addition to a supervised program, unsupervised home AT was also included^45^. Please refer to Table 2 for more details.

**Table 2:** FITT-VP parameters and means of the physical exercise program and control group.

Study, year, country	F	I	T/W	T	V	P	P	M	Duration of interventions (weeks)	Supervised exercise	CG
Krogh, 2012 Dinamarca	3	65% VO2MAX	90 min	Aerobic training	30 min	Second month: 70% Third month: 80%	NR	Cycle ergometer	12	Yes	Stretching exercise
Krogh, 2014 Dinamarca	3	80% HRmax	135 min	Aerobic training	45 min	NR	NR	Cycle ergometer	12	Yes	Stretching exercise
Singh, 1996 USA	3	80% 1RM	180 min	Resistance training	3 sets of 8 repetitions	Each session was tolerated by the subjects	SPE	Exercise machines	10	Yes	No exercise interventions
Herman, 2002 USA	3	70%–85% HRR	90 min	Aerobic training	30 min	NR	SPE	Cycle ergometer, or brisk walking or jogging	16	Yes	Medication (sertraline 100 mg)
Blumenthal, 1999 USA	3	70%–85% HRR	90 min	Aerobic training	30 min	NR	SPE	Cycle ergometer, or brisk walking or jogging	16	Yes	Medication (sertraline 50–200 mg)
Khatri, 2001 USA	3	70%–85% HRR	90 min	Aerobic training	31 min	NR	SPE	Cycle ergometer, or brisk walking or jogging	16	Yes	Medication (sertraline 50–200 mg)
Blumenthal, 2007 USA	3	70%–85% HRR	90 min	Aerobic training	30 min	NR	SPE	Walking or jogging on a treadmill	16	Yes	Medication (sertraline 50–200 mg)
Blumenthal, 2007 USA	3	70%–85% HRR	90 min	Home-based aerobic training	30 min	NR	SPE	Walking or jogging on a treadmill	16	No	Medication (sertraline 50–200 mg)
Sadeghi, 2016 Iran	NR	60–80% HRmax	NR	Aerobic training	30 min	NR	NR	Running in place	8	Yes	12 sessions of cognitive behavior therapy
Dunn, 2005 USA	3	Low intensity	90 min	Aerobic training	30 min	NR	NR	Treadmill or stationary bicycle	12	Yes	Stretching flexibility
Dunn, 2005 USA	5	Low intensity	150 min	Aerobic training	30 min	NR	NR	Treadmill or stationary bicycle	12	Yes	Stretching flexibility
Dunn, 2005 USA	3	High intensity	90 min	Aerobic training	30 min	NR	NR	Treadmill or stationary bicycle	12	Yes	Stretching flexibility
Dunn, 2005 USA	5	High intensity	150 min	Aerobic training	30 min	NR	NR	Treadmill or stationary bicycle	12	Yes	Stretching flexibility

*F* frequency, *I* intensity, *RM* repetition maximum, *T/W* total working time per week, *min* minute, *T* type of exercise, *V* volume, *P* progression, *M* means of exercise, *CG* control group, *NR* not reported, *SPE* Subjective perception of effort, *VO2MAX* Maximum Oxygen Consumption, *HRmax* Maximum heart rate, *HRR* Reserve heart rate.

#### AT (8RCTs)

The AT programs included in the studies varied in frequency, duration, and intensity. The programs were typically conducted three to five days a week, with a duration ranging from eight to 16 weeks. Each session lasted between 30 to 45 min. The intensity of the AT interventions was determined using different parameters.

Some studies reported using minimum intensities of 60% and maximum intensities of 80% based on maximum heart rate (HRMAX) (two studies, 22.22%)^39,40^. Another study used an intensity of 65% of maximum oxygen consumption (VO2 max) (one study, 11.11%)^41^. Four studies (44.44%) by Herman (2002), Blumenthal (1999), Khatri (2001), and Blumenthal (2007) implemented AT with intensities ranging from 70 to 85% of reserve heart rate (RHR)^42–45^. One study categorized intensity into low and high categories (one study, 11.11%)^46^. The total volume of AT per week ranged from 90 to 180 min.

Only one study reported exercise progression, with the intensity increasing from 70% in the second month to 80% in the third month^41^. Some studies based the progression of exercise on subjective perception of effort (four studies, 44.44%)^42–45^. However, several studies did not provide details on the extent of this progression or the timing of the interventions^39–41,46^.

The modes of exercise used in the AT interventions included the cycle ergometer (two studies, 22.22%)^39,41^, a combination of cycle ergometer, fast gait or jogging (two studies, 22.22%)^42,44^, walking or jogging on a treadmill (one study, 11.11%)^45^, running in the same place (one study, 11.11%)^40^, and treadmill or stationary bike (one study, 11.11%)^46^. Please refer to Table 2 for more details.

#### RE (one RCT)

This exercise modality was performed with a frequency of three times per week and lasted for 10 weeks. The intensity was set at 80% of the one-repetition maximum (1RM), and each exercise consisted of three sets of eight repetitions. The progression depended on the individuals' tolerance capacity, and the decision for progression was based on their subjective perception of effort. The resistance exercises targeted large muscle groups using machines such as chest press, overhead pulldown, leg press, knee extension, and flexion. Each session lasted one hour and was accompanied by five minutes of stretching^47^, Table 2*.*

### Comparisons reported in the included studies

Three studies (33.33%) compared AT with flexibility exercise^39,41,46^. In one trial that implemented RE (11.11%), the control group did not undergo any type of intervention^47^. Many of the studies used medication, primarily sertraline, as the comparator (five studies, 55.55%)^42–45^. Only one trial (11.11%) reported the use of cognitive behavioral therapy^40^, Table 2*.*

### Methodological quality evaluation

Few studies in this systematic review had methodological difficulties (mean score 12). The quality assessment results are presented in Supplement 1. Two RCTs (22.22%) did not specify the method used for participant randomization^40,47^. Six studies (66.66%) did not describe whether group allocation was concealed from eligible patients^39–41,43,44,47^. One RCTs (11.11%) did not report if the assessor of at least one primary outcome measure was blinded to group assignment^40^. Five studies (55.55%) did not report adherence rates above 85%^39,40,42,43,45^. Two trials (22.22%) reported no adverse events related to the interventions^39,40^. Two RCTs (22.22%) did not report individual participation in exercise programs^40,44^. Four studies (44.44%) did not perform intention-to-treat analyses for the outcomes of interest^40,42,44,47^. Two studies (22.22%) did not provide point estimates in their results^43,45^, and two did not collect information on physical activity levels from the control group^39,40^. Lastly, one RCTs (11.11%) did not calculate exercise volume and energy expenditure^40^. More details on the quality assessment are presented in Supplement 1.

### Risk of bias of individual studies

Four studies (44.44%) achieved a low risk of bias in the domain evaluating the randomization process^39,41,45,46^. In one RCT (11.11%), the same rating was observed due to deviations from the planned interventions^45^. On the other hand, eight trials (88.88%) were rated as low risk of bias in terms of incomplete outcome data^39,41–47^. Five studies (55.55%) achieved a low risk of bias in outcome measurement^39,41–43,45^. Only three RCTs (33.33%) demonstrated a low risk of bias in reported outcome selection^39,41,45^. Overall, only one study (11.11%) received a low risk of bias rating^45^. Meanwhile, six trials (66.66%) were assessed as having a high risk of bias^40,42–44,46,47^, and two (22.22%) were rated as having some concerns^39,41^. Please refer to Supplement 1 for more details.

### Report of exercise interventions in physical exercise programs

The reporting of CERT elements varied widely among the interventions, ranging from 0 to 100%. The most reported items were exercise supervision or lack thereof, detailed description and characteristics of exercises and interventions for replication purposes, adaptation, and initial level of PE programs for individuals, and whether there were any changes in planned exercises (reported in 100% of the interventions, 13 out of 13). On the other hand, the least reported items were motivation strategies (0.0%), qualifications and experience of those conducting the interventions, progression, and environment where exercise programs were conducted (reported in five interventions, 38.46%), and the description of any component at home or activities other than PE (reported in two interventions, 15.38%). Six CERT elements had reporting rates between 0 and 50% (items 2, 6, 7, 9, 10, 12), while six items were reported in 100% of the interventions (items 8, 13–16). Please refer to Supplement 1 for more information.

### Evidence summary

#### Qualitative synthesis

In the nine included RCTs, the effect of PE interventions on MDD symptomatology was investigated and compared to second-generation antidepressants or cognitive behavioral therapy, BME or no exercise interventions^39–47^. Out of these studies, four reported greater reductions in MDD symptomatology scores because of the PE interventions^42,45–47^. Conversely, four trials reported lower results in favor of the comparators (medication and BME)^39,41,43,44^. One study conducted by Sadeghi in 2016^40^ included three groups: AT, cognitive behavioral therapy, and no exercise interventions. At the end of the eight-week, lower scores were found in participants who were part of the AT and psychotherapy group, indicating a positive effect of both interventions on MDD symptomatology.

#### Adverse events summary

One out of seven RCTs (14.2%) reported no adverse events (AEs) during the follow-up period. However, this study specifically mentioned that AEs were caused by performing the oxygen consumption test^44^.

On the other hand, six out of seven studies (85.7%) evaluated and reported AEs^41–43,45–47^. Among these six trials, 5^41–43,45,46^ mentioned the occurrence of participant withdrawals due to medical or health-related reasons.

#### Adverse events in aerobic exercise participants

Among the 375 participants in the PE interventions, a total of 25 AEs were reported. These events were classified as grade one (5 events), grade two (10 events), grade three (9 events), grade four (1 event), and grade five (0 events). However, it should be noted that 19 of these events were not related to the intervention.

Among the participants in the PE group, six AEs (31%) occurred. These AEs included muscular events, other painful manifestations, and a medical contraindication. It is worth mentioning that these events were managed by using the cycle ergometer as the primary means of PE and additional medical review. For more details, please refer to Supplement 1.

Adverse events in participants on second-generation antidepressants, BME or no exercise interventions.

Among the participants receiving second-generation antidepressants, BME or no exercise interventions. a total of 39 AEs were reported among 308 participants. These events were classified as grade one (0 events), grade two (14 events), grade three (25 events), grade four (0 events), and grade five (0 events).

Out of the 39 reported AEs, 21 (54%) were related to medications. These medication-related AEs included symptoms such as dizziness, drowsiness, agitation, and diarrhea. For more information, please refer to Supplement 1.

### Quantitative synthesis

#### Primary outcome: depressive symptoms

**Figure 2:**
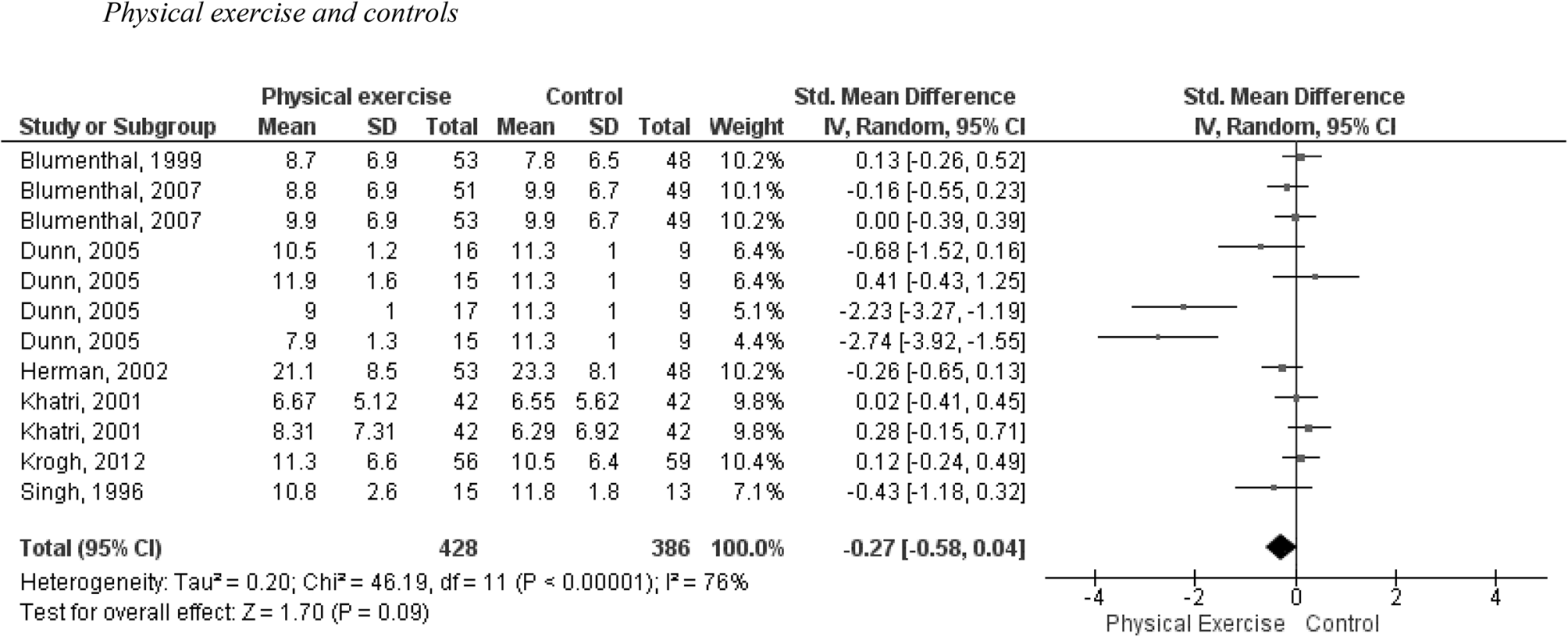
Analysis of the effect of PE programs on depressive symptomatology in adults with MDD compared to control. I^2^: heterogeneity, *p* value < 0.05, standardized mean difference, Random effects model.

Figure 2 displays the results of the meta-analysis, which compares the effects of exercise modalities (AT, RE) with second-generation antidepressants, BME or no exercise interventions on the symptoms of MDD. The forest plot does not present the results of AEs since they are assessed using a different approach.

The meta-analysis included 7 trials with a total of 12 interventions. The pooled SMD, calculated using the random effects model, was -0.27 with a 95% CI of [-0.58, 0.04). This indicates a small clinical effect in favor of exercise interventions, although the difference was not statistically significant. It is important to note that there was considerable heterogeneity among the included studies, as indicated by an I^2^ value of 76%.

#### Subgroup analysis

The analysis of specific subgroups and comparisons within the review provides additional insights into the effects of PE interventions on MDD symptoms. When focusing on RE, there was a non-significant small effect (− 0.43) observed in one study, indicating a potential benefit but not reaching statistical significance (P = 0.26). Similarly, when using the HAM-D, a small effect (− 0.46) was found, which approached statistical significance (P = 0.06). However, both analyses showed high heterogeneity (83% and 82% respectively).

A subgroup analysis targeting overweight and obese adults (one study) revealed a large effect size (− 1.27), although it did not reach statistical significance (P = 0.07). The analysis based on age (two studies) showed a large effect (− 0.94) in individuals under 50 years old, but again, statistical significance was not achieved (P = 0.09). Furthermore, PE performed five days per week (one study) demonstrated a large effect size (− 1.13), but with considerable heterogeneity (94%). From one trial of multiple interventions, high-intensity PE interventions displayed a greater effect size (− 2.45) compared to the primary outcomes, and it was statistically significant (P < 0.00001), with no heterogeneity observed (heterogeneity = 0%).

In terms of comparisons with different control conditions, when comparing PE (mainly AT) with medication, no significant effect size was found (− 0.01, P = 0.94), with low heterogeneity (0%). Conversely, when comparing PE with flexibility exercise, a large effect size (− 0.94) was observed, but it did not reach statistical significance (P = 0.09) and showed high heterogeneity (90%). Only one study compared PE (RE) with no exercise, resulting in a non-significant effect size (− 0.43, P = 0.26).

Please refer to Supplement 1 for further details on these findings.

### Sensitivity analysis

In this review, this analysis was proposed using studies with controls without exercise interventions and a low risk of bias in the domains related to the randomization process and deviations from the intended interventions. Blumenthal et al.^45^ was the only study that obtained a low risk of bias rating in both domains. However, 4 studies were judged to have a low risk of bias in the randomization process^39,41,45,46^. However, Krogh et al.^39^ was not included in the meta-analyses due to its lower methodological quality rating.

**Figure 3:**
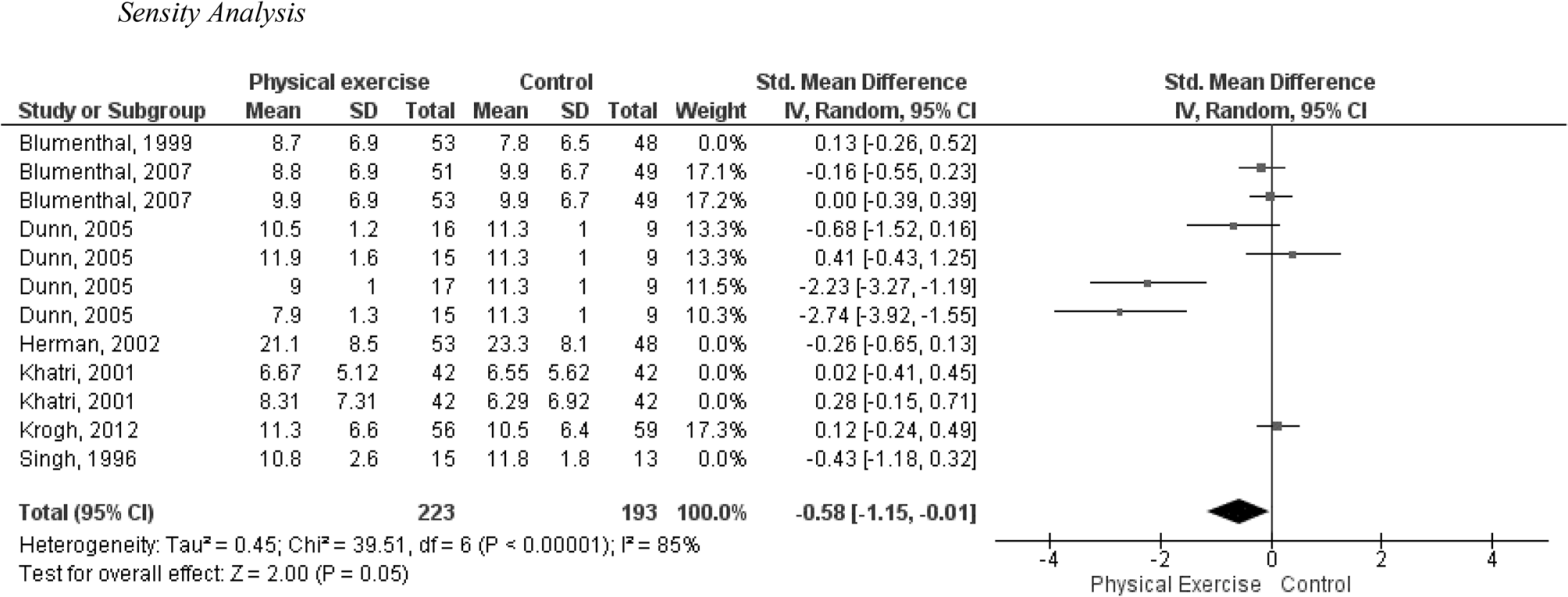
Sensitivity analysis by removing studies with high risk of bias. I^2^: heterogeneity, *p* value < 0.05, standardized mean difference, Random effects model.

The analysis of these selected studies showed a moderate effect size in favor of PE compared to the primary results, with an effect size of -0.58. This effect size was not statistically significant, as indicated by the test for overall effect (Z = 2.00, P = 0.05). It is important to note that the level of imprecision and heterogeneity in these results was considerable. The estimated Tau^2^ was 0.45, the Chi^2^ value was 39.51 with degrees of freedom (df) = 6 (P < 0.00001), and the I^2^ value was 85%. Please refer to Fig. 3 for a visual representation of these findings.

Meta-analyses of the non-exercise interventions control study Singh et al.^47^ reported similar findings to the primary outcomes, with an effect size of -0.26 (95% CI − 0.59, 0.07, I^2^ = 78%, P = 0.12). This suggests that no significant differences were found between interventions and controls in terms of depressive symptoms when comparing BME and medication (sertraline).

#### Adverse events

**Figure 4:**
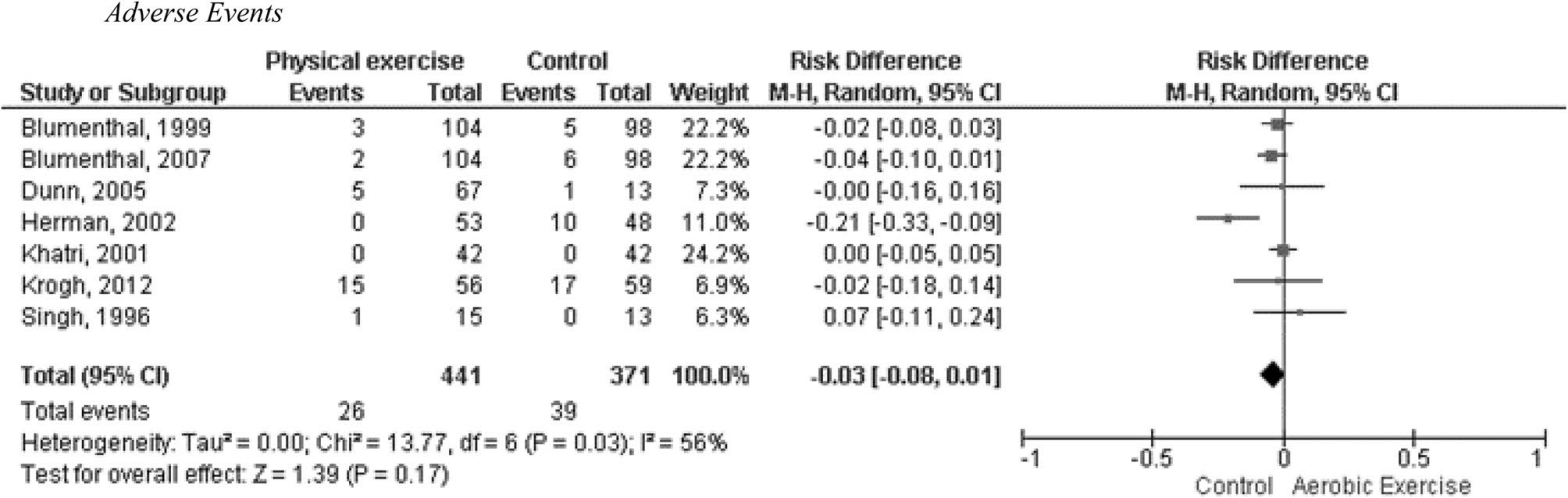
Analysis of the effect of PE programs on AEs in adults with MDD compared to control. I^2^: heterogeneity, p value < 0,05. Risk difference. Mantel–Haenszel random effects model.

The pooled analysis of seven randomized controlled trials (13 exercise arms) involving 812 participants (PE: n = 441; BME: n = 371) did not find a significant difference in the risk of grade one to five AEs between PE interventions and second-generation antidepressants, BME or no exercise interventions. The analysis included a total of 65 AEs. The RD was − 0.03 with a 95% CI ranging from − 0.08 to 0.01. The P-value was 0.17, indicating no statistically significant difference. The I^2^ value was 56%, suggesting moderate heterogeneity among the studies. Please refer to Fig. 4 for a graphical representation of these findings.

#### Quality of life

The two studies included in the meta-analysis, Krogh et al.^41^ and Singh et al.^47^, evaluated the effect of PE interventions on general well-being in adults with MDD. Krogh et al. assessed well-being using the Five-Well-Being Index (WHQ-5) and found no statistically significant difference in post-intervention scores between the PE group and the control group (p = 0.74). Singh et al. evaluated well-being using the general health dimension of the health questionnaire (SF-36) and found a borderline significant difference between the intervention and control groups (p = 0.06).

Although there were only two studies with a total of 147 adults, the reviewers decided to conduct a meta-analysis. The pooled analysis showed a small effect size that slightly favored the control group, with a standardized mean difference of − 0.04. However, this effect size was not statistically significant (p = 0.79), indicating no significant difference in general well-being between the PE interventions and the control conditions. The heterogeneity among the studies was very low (I^2^ = 0%), suggesting consistency in the results. Please refer to Supplement 1 for further details.

#### Mortality

In the nine studies included in the review, no deaths were reported among any of the participants^39–47^.

### Certainty of the evidence (GRADE)

The overall quality and certainty of the evidence in this review ranged from very low to low. The main factors contributing to the downgrade in quality were the risk of bias and imprecision, which were related to methodological shortcomings in the included RCTs and wide confidence intervals. Here are the key findings based on the level of evidence:

- *Depressive symptoms* There is very low-quality evidence suggesting a potential reduction in depressive symptoms when comparing PE to second-generation antidepressants, BME or no exercise interventions. The SMD was − 0.27 with a 95% CI of [− 0.58, 0.04]. However, the statistical significance was not reached (P = 0.09), and there was considerable heterogeneity (I^2^ = 76%). The evidence was downgraded due to risk of bias, inconsistency, and imprecision.
- *AEs* There is low-quality evidence indicating no significant difference in the risk of grade 1 to 5 AEs between PE and second-generation antidepressants, BME or no exercise interventions. The RD was − 0.03 with a 95% CI of [− 0.08, 0.01]. The statistical analysis showed no significance (P = 0.17), and there was moderate heterogeneity (I^2^ = 56%). The evidence was downgraded only for risk of bias.
- *Quality of life* There is very low-quality evidence suggesting no significant difference in the reduction of quality of life between PE and second-generation antidepressants, BME or no exercise interventions. The SMD was − 0.04 with a 95% CI of [− 0.37, 0.28]. The analysis did not show statistical significance (P = 0.79), and there was no heterogeneity (I^2^ = 0%). The evidence was downgraded for risk of bias, indirect evidence, and imprecision.

Regarding mortality, it could not be estimated as there were no reported deaths in the included studies. Therefore, no conclusions can be drawn regarding the impact of PE interventions on mortality based on the available evidence.

It is important to consider the limitations of the included studies and the overall quality of the evidence when interpreting these findings.

## Discussion

### Summary of main results

In this review, a total of nine trials were included, out of which seven were rated as having high methodological quality (meta-analysis). These trials provided valuable insights into the benefits and potential harms associated with PE (AT and RE) interventions for individuals with MDD without second-generation antidepressants or cognitive behavioral therapy before PE interventions. The study population was heterogeneous, consisting of young and older adults, both sexes, some of whom were overweight or obese and had various comorbidities such as endocrine, cardiac, pulmonary, and orthopedic disorders. Many of the participants were not physically active at baseline.

These trials allowed for comparisons between PE and other interventions such as medication use, flexibility exercise, cognitive behavioral therapy, or no exercise intervention. The main findings indicate that supervised PE, primarily AT, had a small effect size in reducing depressive symptoms compared to control conditions groups, although the difference was not statistically significant.

Some of the subgroup analyses showed a large effect (overweight and obese adults, individuals under 50 years old, five days per week, and comparing PE with flexibility exercise). However, it is important to note that many of these effects are based on findings from one or two trials, and when compared with control conditions, no statistically significant differences and considerable heterogeneity was observed. Only the supervised PE performed at high intensities (one study with multiple interventions) had a large effect size, presenting significant differences and 0% heterogeneity.

In the sensitivity analyses, moderate and small effects were observed, although they did not show statistically significant differences when performed by selecting low risk of bias in the domains related to the randomization process and deviations from the intended interventions and non-exercise interventions control study.

Regarding safety, 1 RCT^41^ provided inconclusive evidence on the safety of exercise interventions. 19 reported AEs were not directly related to PE, and some of them were managed with the use of a cycle ergometer. There was a higher incidence of AEs reported in control groups receiving medication, including symptoms such as dizziness, drowsiness, agitation, and diarrhea. The effect of PE on quality of life, based on data from 147 adults, was rated as trivial and did not reach statistical significance. Lastly, none of the included studies reported any instances of mortality among the participants.

### Overall completeness and applicability of evidence

The findings of this review primarily apply to adults between the ages of 20 and 72 who were diagnosed with MDD and were not receiving second-generation antidepressants or cognitive behavioral therapy. However, it's important to note that some of the included RCTs did not provide sufficient information about the participants' characteristics, such as body composition or the use of medications for other conditions. Additionally, information about the participants' usual or non-usual physical activity levels and employment status was not consistently reported across all trials.

To the best of the reviewers' knowledge, this is the first systematic review to specifically evaluate the effects of PE on MDD symptoms in individuals without prior use of second-generation antidepressants or cognitive behavioral therapy. The subgroup analyses conducted in the review provide detailed insights into the effects of PE compared to control conditions on specific symptoms associated with MDD. This level of detail enhances our understanding of each intervention and its impact on the symptoms of the disorder.

### Certainty of the evidence

Indeed, the strength of evidence in this review (GRADE framework) was generally assessed as low to very low. This assessment was influenced by several limitations identified in the included studies, including issues related to risk of bias, inconsistency, and imprecision.

While seven out of the nine included RCTs were evaluated as high quality, some of them had certain shortcomings. For instance, they did not publish the trial protocols in a designated repository and failed to provide detailed descriptions of the statistical or mathematical procedures used for sample size calculations.

The overall risk of bias for the included RCTs was determined to be high, with some concerns. This was primarily due to a lack of reporting regarding the methods used for implementing and concealing randomization, absence of blinding of participants, intervention providers, and outcome assessors, as well as deviations from the intended interventions in some cases.

Additionally, few RCTs provided information on deviations from the planned interventions resulting from the trial setting. These limitations contribute to the overall assessment of the quality of evidence and highlight the need for more rigorous study design and reporting in future research.

### Potential biases in the overview process

This review has some limitations, the first having to do with clinical, methodological, and statistical heterogeneity. From a clinical point of view, the effect of PE was affected by the characteristics of the patients, the interventions, and the results. The true effect of the intervention differed between studies. In methodological terms, some studies do not report blinding and concealment of the allocation sequence, and different instruments were used to measure depressive symptomatology. In summary, the studies suffered from different degrees of bias. Also, in the estimated effect, we obtained a large Chi^2^ (statistical test of heterogeneity) and a small P value, which translates into heterogeneity of the effects of the interventions. This is because the review had few studies and small sample sizes (uncertainty in the I^2^ value). Therefore, the presence of heterogeneity affected the extent to which generalizable conclusions can be made. Although we performed an analysis based on random effects, these results need to be taken with caution. On the other hand, we ran a subgroup analysis to explore heterogeneity. There we find that it was substantial and considerable. This further strengthens the cautious interpretation of these findings. By excluding from the meta-analysis two atypical interventions from the study by Dunn et al.^46^ (Frequency 3; High intensity; session duration between 90 and 150 min for 12 weeks), we found an effect size of − 0.01 [− 0.16, 0.14] I^2^ = 6% (P = 0.39), which evidently demonstrated that the results of these interventions conflicted with the rest of the studies.

The second limitation of this review has to do with the lack of statistical power. An example of this is the subgroup analyses since we have less than 10 studies for each characteristic chosen for the analysis. In summary, this review not only has fewer than 10 included studies but also fewer included studies than analyzes performed.

A potential strength of this review was the performance of random-effects meta-analyses of continuous data (different, but related studies), because the outcome was measured using different scales or units. This model assumes that the differences observed between the results of the RCTs are due to a combination of chance and some genuine variation in the effects of the intervention. We also used the SMD, a recommended test to avoid extreme heterogeneity in the results when cases such as the one described above occur. But the random effects model also has a drawback in interpreting these findings. This is because, in the presence of heterogeneity, it gives greater statistical weight to studies with small effects and less weight to those with large effects. Added to this is the asymmetry between the studies (imprecision), which possibly pushed the results of the randomized model towards the findings of the smaller studies.

Indeed, the lack of detailed information on the progression and decision-making related to PE programs is a notable limitation of this review. Without clear and comprehensive descriptions of how the PE interventions were implemented, it becomes challenging for healthcare professionals to replicate and apply these interventions in clinical practice effectively.

On the other hand, this systematic review adhered to the highest methodological standards, following established guidelines^20,22,30^. Rigorous methods were employed, including comprehensive searches of scientific databases, clinical trial repositories, grey literature, and manual searches. The process of study selection and data extraction was carried out independently by reviewers who were blinded to minimize bias. This robust methodology strengthens the reliability and credibility of the study.

The experience and expertise of the research team, including university professors and physicians specialized in exercise science and physical activity, undoubtedly constitute a significant strength of this review. However, as the authors themselves acknowledge, the absence of a psychiatrist or mental health expert within the group of reviewers is an important limitation.

The findings and certainty of evidence generated by this study will serve as a valuable resource for the development of future clinical practice guidelines, particularly those focusing on non-pharmacological strategies for the treatment of depressive disorders. However, it is important to acknowledge the methodological limitations identified in the included RCTs. These limitations should be taken into consideration when interpreting the results and applying them to clinical practice.

### Agreements and disagreements with other reviews

In recent years, systematic reviews have been published examining the effect of PE on adults with and without MDD^2–4,12,16,48–51^. These reviews generally support the notion that exercise can reduce the symptoms of MDD. However, it is worth noting that some of these reviews did not use the GRADE framework to evaluate and classify the certainty of their findings^2,12,16,48^, and the level of certainty in others varied from moderate to very low quality^2–4,49,51^.

One systematic review^4^ found a larger effect size than the present review, but this effect was only significant when compared to no intervention or placebo. Additionally, their review did not assess the effect of PE compared to all types of controls, including placebo, psychological therapy, alternative treatments, and medications, among others.

There are other systematic reviews that have reported findings like our study^2,16,49^. For example, Krogh et al. in^49^, reported a trivial effect size of very low quality when pooling studies with low risk of bias. They also found it challenging to assess adverse events due to a lack of information. In our review, we observed a low effect size based on studies of high methodological quality (TESTEX). Furthermore, our findings suggest that PE is associated with fewer harms compared to medications.

Seshadri et al. in^2^ examined the effects of various forms of exercise (including PE, yoga, and Tai chi) on reducing depressive symptoms in adults with MDD. Some of the included studies in their review involved participants who were concurrently using medication. However, our review did not include exercise as an adjunct to medication but rather compared exercise to other control interventions. Additionally, our review specifically focused on individuals who were not receiving second-generation antidepressants or cognitive behavioral therapy for MDD before PE interventions.

This review employed a specific search strategy guided by the COCHRANE Ibero-America network's expert librarians, resulting in a smaller number of RCTs to screen compared to previous publications. By following the established guidelines and utilizing accurate mapping of studies using Medical Subject Headings (MeSH), we ensured the inclusion of all relevant RCTs related to our PICOTS.

In contrast, Krogh et al.^49^ screened a larger number of trials (25,435) due to different search terms used in their search strategy. Furthermore, their study did not specifically include the term "Depressive Disorder, Major," which is crucial for identifying clinical or major depression. Additionally, their review included RCTs comparing exercise interventions with controls and exercise plus medication with controls. However, it is not specified whether the patients in their included studies were already undergoing second-generation medication or cognitive behavioral therapy before the exercise interventions.

Similarly, Yu et al.^51^ reported a larger number of studies in their search results because they aimed to cover various mental health disorders, including depression, anxiety, phobias, post-traumatic stress, mood disorders, among others.

Another study by Wu et al. in 2023 investigated the effects of Yoga interventions on the severity of symptoms associated with MDD^52^. Their findings indicated a moderate effect, but the certainty of evidence was rated as low to moderate. It is important to note that some of the RCTs included in their review incorporated second-generation antidepressants or cognitive behavioral therapy alongside Yoga interventions. They performed subgroup analyses considering factors such as patients' place of residence, duration of interventions, frequency, and whether the intervention was performed independently or combined with meditation. However, subgroup analyses comparing the interventions to control conditions were not reported.

In contrast, our review specifically focused on the effects of exercise interventions in individuals who were not receiving second-generation antidepressants or cognitive behavioral therapy prior to the interventions. This was considered a comparator in our study. In our subgroup analysis, when comparing AT with medication (sertraline), the effect size was not statistically significant (− 0.01 [− 0.17, 0.16], I^2^ = 0%). This suggests that both treatments may be effective in improving symptoms.

However, it is important to note that this comparison was specific to sertraline, and the effectiveness of exercise compared to other medications such as escitalopram, citalopram, fluoxetine, paroxetine, or duloxetine remains unclear. Considering patient preferences is important, as medications may have adverse events and may be difficult to access in low- and middle-income countries^14,53^. On the other hand, exercise is a safe and easily accessible non-pharmacological treatment option for various populations.

In our sensitivity analyses, we observed a different effect size when compared to the control groups. These results suggest that, when considering studies with a low risk of bias in important methodological aspects (RoB II), there is a moderate effect size favoring PE interventions in reducing symptoms of MDD. However, it is important to note that this result did not reach statistical significance. Furthermore, the presence of high heterogeneity and imprecision among the included studies emphasizes the need for further research and investigation to better understand the true impact of PE interventions on MDD symptoms. Caution should be exercised when interpreting these findings.

### Implications for practice

This systematic review offers a comprehensive and current overview of the impact of PE on MDD in adults who have not received second-generation antidepressants or cognitive behavioral therapy prior to exercise interventions. The findings of this review can be valuable for individuals and their families affected by depression, general practitioners, psychiatrists, professionals in the field of physical activity, and policymakers involved in mental health. However, it should be noted that some of the included RCTs lack complete information on the PE programs, which may hinder their replication in interested communities.

The results of this review suggest that there are some promising effects observed in certain subgroups when it comes to the impact of PE interventions on depressive symptoms in adults with MDD. However, it is crucial to approach these findings with caution, considering the limitations of the included studies and the potential heterogeneity among them. The overall evidence from the review is inconclusive, mainly due to the risk of bias of the studies. The small number of trials and participants included in some subgroups may have affected the statistical power and precision of the results. Therefore, the lack of statistical significance in certain subgroups should be interpreted with caution, as it may be influenced by the limited sample size.

In conclusion, while this review offers valuable insights into the potential effects of PE interventions on depressive symptoms in adults with MDD, it also highlights the need for further research to address the limitations and strengthen the evidence.

### Implications for future research

It is currently not possible to definitively determine the optimal dose of PE required to reduce depressive symptoms in patients with MDD who do not receive second-generation drugs or attend cognitive behavioral therapy.

Future RCTs should be conducted with homogeneous populations, considering detailed and precise definitions of the characteristics of exercise interventions. Specifically, they should assess the effect when exercise is performed at moderate intensities compared to high intensities.

Additionally, it is important to explore the effects of exercise modalities other than AT. Considering RE and its combination with AT for populations with this disorder is crucial. Similarly, these studies should involve multidisciplinary researchers, including psychiatrists and physical educators. Furthermore, future research should place a strong emphasis on providing comprehensive and detailed descriptions of PE interventions, including their progression and decision-making processes. This will enhance the transparency and reproducibility of the studies and enable clinicians to implement evidence-based exercise interventions with greater confidence in managing depressive symptoms in individuals with MDD.

Likewise, the results of this review suggest that future RCTs should be developed in full compliance with protocol construction checklists^54^ and final reports on non-pharmacological randomized controlled trials^55^. Furthermore, it is important to evaluate the methodological quality of these studies using instruments specifically designed for this purpose, such as the TESTEX^29^. Detailed descriptions of exercise interventions should be provided to facilitate replication. Authors should adhere to international reporting guidelines, such as the Consensus on Exercise Reporting Template (CERT)^31^ or the Intervention Description and Replication Template (TIDieR) checklist and guide^56^, when formulating and publishing these studies. However, it should be noted that some RCTs included in this review did not publish their protocols in a controlled trial repository. To address this issue, adherence to the CONSORT Statement^55^ is recommended. Future systematic reviews focusing on this topic should assess the strength and certainty of the results^20^ to ensure credibility for decision-makers.

Therefore, considering the limitations identified in this review, further research is needed to provide a clearer understanding of the effects of PE interventions on depressive symptoms in individuals with MDD. Larger-scale, well-designed RCTs with consistent methodologies are necessary to establish stronger and more reliable evidence in this area.

## Conclusion

The available evidence, although of low to very low certainty, indicates that supervised PE (mainly AT) does not show statistically significant differences when compared with second-generation medication or cognitive behavioral therapy, BME, or no exercise interventions in terms of managing symptoms caused by MDD. Additionally, no significant differences were observed in terms of harm or adverse events between these interventions. Subgroup and sensitivity analyses showed moderate and large effects in favor of PE, but without statistical significance and with high heterogeneity.

Indeed, it is crucial to interpret these results with caution due to the limitations mentioned earlier in this review. The identified limitations, such as clinical, methodological, and statistical heterogeneity among the included studies, small sample sizes, and lack of detailed information on progression and decisions related to PE programs, may impact the generalizability and applicability of the findings.

### Supplementary Information

Supplementary Information.

## Data Availability

The data sets generated and/or analyzed during the implementation of the study are available from the corresponding author upon request.
