# Supplementary material for: Physical exercise and major depressive disorder in adults: systematic review and meta-analysis
Source: Sci Rep. 2023 Aug 14;13:13223. doi: 10.1038/s41598-023-39783-2 (PMC10425328; doi:10.1038/s41598-023-39783-2)
Supplement: Supplementary file 1 — Supplementary Information. [file 41598_2023_39783_MOESM1_ESM.pdf]

PHYSICAL EXERCISE AND MAJOR DEPRESSIVE DISORDER IN ADULTS: SYSTEMATIC REVIEW AND META-ANALYSIS.

PhD. Édison Andrés Pérez Bedoya. Department of Physical Education of the Federal University of Viçosa, Minas Gerais, Brazil. Email: edison.bedoya@ufv.br. ORCID: 0000-0002-1929-9132

MSc. Luisa Fernanda Puerta López. Department of Physical Education of the Antioquia University, Medellín, Colombia. Email: luisa.puerta1@udea.edu.co ORCID: 0000-0001-6177-1456

BSc. Daniel Alejandro López Galvis. Department of Physical Education of the Antioquia University, Medellín, Colombia. Email: daniel.lopez12@udea.edu.co ORCID: 0000-0003-1164-0639

PhD. Diego Alejandro Rojas Jaimes. Department of Physical Education of Llanos University, Villavicencio, Colombia. Email: diego.rojas.jaimes@unillanos.edu.co ORCID: 0000-0002-4133-1653

PhD. Osvaldo Costa Moreira. Department of Physical Education of the Federal University of Viçosa, Minas Gerais, Brazil. Email: osvaldo.moreira@ufv.br ORCID: 0000-0002-1386-8883

Contact information for the corresponding author.

Édison Andrés Pérez Bedoya. Department of Physical Education of the Federal University of Viçosa, Minas Gerais, Brazil. Visiting address: Av. Peter Henry Rolfs, s/n - Campus Universitário, Viçosa - MG, 36570-900 Tel: (+55) 31 972573537. Email: **edison.bedoya@ufv.br**

SEARCH STRATEGY

MEDLINE (via OVID)

Hits: **307**

No restriction on timeline

Medline was searched with appropriate Medical Subject Headings (MeSH). A filter wasestablished: randomized controlled trials.

1	exp " Depressive Disorder, Major "/	38298
2	Depression, Involutional/	38298
3	Major Depressive Disorder Melancholia/	0
4	Involutional Paraphrenia/	38298
5	Involutional Psychosis/	38298
6	Involutional/	0
7	1 or 2 or 3 or 4 or 5 or 6	38298
8	exp " Resistance Training "/	12229
9	Training, Resistance/	12229
10	Strength Training/	12229
11	Training, Strength/	12229
12	Weight-Lifting Strengthening Program/	12229
13	Strengthening Program, Weight-Lifting/	12229
14	Strengthening Programs, Weight-Lifting/	12229
15	Weight Lifting Strengthening Program/	12229
16	Weight-Lifting Strengthening Programs/	12229
17	Weight-Lifting Exercise Program/	12229
18	Exercise Program, Weight-Lifting/	12229
19	Exercise Programs, Weight-Lifting/	12229
20	Weight Lifting Exercise Program/	12229
21	Weight-Lifting Exercise Programs/	12229
22	Weight-Bearing Strengthening Program/	12229
23	Strengthening Program, Weight-Bearing/	12229
24	Strengthening Programs, Weight-Bearing/	12229
25	Weight Bearing Strengthening Program/	12229
26	Weight-Bearing Strengthening Programs/	12229
27	Weight-Bearing Exercise Program/	12229
28	Exercise Program, Weight-Bearing/	12229
29	Exercise Programs, Weight-Bearing/	12229
30	Weight Bearing Exercise Program/	12229
31	Weight-Bearing Exercise Programs/	12229
32	exp "Exercise"/	245608
33	Acute Exercise/	141099
34	Aerobic Exercise/	141099

35 Exercise Training/ 141099

36 Exercise, Aerobic/ 141099

37 Exercise, Physical/ 141099

38 8 or 9 or 10 or 11 or 12 or 13 or 14 or 15 or 16 or 17 or 18 or 19 or 20 or 21 or 22 or 22 or 23 or 24 or 25 or 26 or 27 or 28 or 29 or 30 or 31 or 32 or 33 or 34 or 35 or 36 or 37 245608

39 7 and 38 **307**

CENTRAL

Hits: **765**

No restriction on timeline

CENTRAL was searched with appropriate Medical Subject Headings (MeSH). A filter was established: randomized controlled trials.

ID	Search	Hits
#1	Depressive Disorder, Major	15882
#2	Depression, Involutional	53
#3	Major Depressive Disorder Melancholia	128
#4	Involutional Paraphrenia	2
#5	Involutional Psychosis	11
#6	Involutional	167
#7	#1 OR #2 OR #3 OR #4 OR #5 OR #6	16023
#8	Resistance Training	17212
#9	Training, Resistance	17212
#10	Strength Training	20998
#11	Training, Strength	20998
#12	Weight-Lifting Strengthening Program	43
#13	Strengthening Program, Weight-Lifting	43
#14	Strengthening Programs, Weight-Lifting	28
#15	Weight Lifting Strengthening Program	81
#16	Weight-Lifting Strengthening Programs	28
#17	Weight-Lifting Exercise Program	349
#18	Exercise Program, Weight-Lifting	349
#19	Exercise Programs, Weight-Lifting	135
#20	Weight Lifting Exercise Program	450
#21	Weight-Lifting Exercise Programs	135
#22	Weight-Bearing Strengthening Program	157
#23	Strengthening Program, Weight-Bearing	157
#24	Strengthening Programs, Weight-Bearing	81

#25 Weight Bearing Strengthening Program 168

#26 Weight-Bearing Strengthening Programs 81

#27 Weight-Bearing Exercise Program 557

#28 Exercise Program, Weight-Bearing 557

#29 Exercise Programs, Weight-Bearing 180

#30 Weight Bearing Exercise Program 604

#31 Weight-Bearing Exercise Programs 180

#32 Exercise 127500

#33 Acute Exercise 12622

#34 Aerobic Exercise 17187

#35 Exercise Training 44613

#36 Exercise, Aerobic 17187

#37 Exercise, Physical 57123

#38 #8 OR #9 OR #10 OR #11 OR #12 OR #13 OR #14 OR #15 OR #16 OR #17 OR #18 OR #19 OR #20 OR #21 OR #22 OR #22 OR #23 OR #24 OR #25 OR #26 OR #27 OR #28 OR #29 OR #30 OR #31 OR #32 OR #33 OR #34 OR #35 OR #36 OR #37 135999

#39 #7 AND #38 **765**

Embase

Hits: **1.186**

No restriction on timeline

Embase was searched with appropriate Medical Subject Headings (MeSH). A filter was established: randomized controlled trials.

No. Query Results	Results
#38. #7 AND #37	**1,186**
#37. #8 OR #9 OR #10 OR #11 OR #12 OR #13 OR #14 OR #15 OR #16 OR #17 OR #18 OR #19 OR #20 OR #21 OR #22 OR #23 OR #24 OR #25 OR #26 OR #27 OR #28 OR #29 OR #30 OR #31 OR #32 OR #33 OR #34 OR #35 OR #36	438,192
#36. 'exercise, physical'	1,114
#35. 'exercise, aerobic'/de	22,500
#34. 'exercise training'/de	351,342
#33. 'aerobic exercise'/de	22,500
#32. 'acute exercise'/de	22
#31. 'exercise'/exp	437,805

#30. 'weight-bearing exercise programs'	4
#29. 'weight bearing exercise program'	28
#28. 'exercise programs, weight-bearing'	3
#27. 'exercise program, weight-bearing'	3
#26. 'weight-bearing exercise program'	28
#25. 'weight-bearing strengthening programs'	1
#24. 'weight bearing strengthening program'	4
#23. 'strengthening programs, weight-bearing'	2
#22. 'strengthening program, weight-bearing'	2
#21. 'weight-bearing strengthening program'	4
#20. 'weight-lifting exercise programs'	1
#19. 'weight lifting exercise program'	2
#18. 'exercise programs, weight-lifting'	2
#17. 'exercise program, weight-lifting'	1
#16. 'weight-lifting exercise program'	2
#15. 'weight-lifting strengthening programs'	2
#14. 'weight lifting strengthening program'	1
#13. 'strengthening programs, weight-lifting'	1
#12. 'strengthening program, weight-lifting'	1
#11. 'weight-lifting strengthening program'	1
#10. 'training, strength'	219
#9. 'training, resistance'	217
#8. 'resistance training'/exp	27,047
#7. #1 OR #2 OR #3 OR #4 OR #5 OR #6	84,170
#6. involutional	2,458
#5. 'involutional psychosis'/de	15
#4. 'involutional paraphrenia'	15
#3. 'major depressive disorder melancholia'	15
#2. 'depression, involutional'/de	338
#1. 'depressive disorder, major'/exp	82,071

PsycINFO

Hits: **123**

No restriction on timeline

PsycINFO was searched with appropriate Medical Subject Headings (MeSH). A filter wasestablished: randomized controlled trials.

123 Results for Any Field: exp " Depressive Disorder, Major" / OR Any Field: "Depression, Involutional" / OR Any Field: "Major Depressive Disorder Melancholia" / OR Any Field: "Involutional Paraphrenia" / OR Any Field: "Involutional Psychosis" / OR Any Field: Involutional/ AND Any Field: exp " Resistance Training" / OR Any Field: "Training, Resistance" / OR Any Field: "Strength Training" / OR Any Field: "Training, Strength" / OR Any Field: "Weight-Lifting Strengthening Program" / OR Any Field: "Strengthening Program, Weight-Lifting" / OR Any Field: "Strengthening Programs, Weight-Lifting" / OR Any Field: "Weight Lifting Strengthening Program" / OR Any Field: "Weight-Lifting Strengthening Programs" / OR Any Field: "Weight-Lifting Exercise Program" / OR Any Field: "Exercise Program, Weight-Lifting" / OR Any Field: "Exercise Programs, Weight-Lifting" / OR Any Field: "Weight Lifting Exercise Program" / OR Any Field: "Weight-Lifting Exercise Programs" / OR Any Field: "Weight-Bearing Strengthening Program" / OR Any Field: "Strengthening Program, Weight-Bearing" / OR Any Field: "Strengthening Programs, Weight-Bearing" / OR Any Field: "Weight Bearing Strengthening Program" / OR Any Field: "Weight-Bearing Strengthening Programs" / OR Any Field: "Weight-Bearing Exercise Program" / OR Any Field: "Exercise Program, Weight-Bearing" / OR Any Field: "Exercise Programs, Weight-Bearing" / OR Any Field: "Weight Bearing Exercise Program" / OR Any Field: "Weight-Bearing Exercise Programs" / OR Any Field: exp " Exercise" / OR Any Field: "Acute Exercise" / OR Any Field: "Aerobic Exercise" / OR Any Field: "Exercise Training" / OR Any Field: "Exercise, Aerobic" / OR Any Field: "Exercise, Physical" /

Web of science

Hits: **34**

No restriction on timeline

Web of science was searched with appropriate Medical Subject Headings (MeSH). A filter wasestablished: randomized controlled trials.

1: (((((ALL=(" Depressive Disorder, Major")) OR ALL=("Depression, Involutional")) OR ALL=("Major Depressive Disorder Melancholia")) OR ALL=("Involutional Paraphrenia")) OR ALL=("Involutional Psychosis")) OR ALL=(Involutional)

Results: 1192

2: ((((((((((((((((((((((((((((ALL=(" Resistance Training")) OR ALL=("Training, Resistance")) OR ALL=("Strength Training")) OR ALL=("Training, Strength")) OR ALL=("Weight-Lifting Strengthening Program")) OR ALL=("Strengthening Program, Weight-Lifting")) OR ALL=("Strengthening Programs, Weight-Lifting")) OR ALL=("Weight Lifting Strengthening Program")) OR ALL=("Weight-Lifting Strengthening Programs")) OR ALL=("Weight-Lifting Exercise Program")) OR ALL=("Exercise Program, Weight-Lifting")) OR ALL=("Exercise Programs, Weight-Lifting")) OR ALL=("Weight Lifting Exercise Program")) OR ALL=("Weight-Lifting Exercise Programs")) OR ALL=("Weight-Bearing Strengthening Program")) OR ALL=("Strengthening Program, Weight-Bearing")) OR ALL=("Strengthening Programs, Weight-Bearing")) OR ALL=("Weight Bearing Strengthening Program")) OR ALL=("Weight-Bearing Strengthening Programs")) OR ALL=("Weight-Bearing Exercise Program")) OR ALL=("Exercise Program, Weight-Bearing")) OR ALL=("Exercise Programs, Weight-Bearing")) OR ALL=("Weight Bearing Exercise Program")) OR ALL=("Weight-Bearing Exercise Programs")) OR ALL=(" Exercise")) OR ALL=("Acute Exercise")) OR ALL=("Aerobic Exercise")) OR ALL=("Exercise Training")) OR ALL=("Exercise, Aerobic")) OR ALL=("Exercise, Physical")

Results: 633203

3: #1 AND #2 **Results: 34**

Clinical Trials

Hits: **14**

No restriction on timeline

Clinical trials were searched with appropriate Medical Subject Headings (MeSH). A filter wasestablished: randomized controlled trials.

**Other terms:**	randomized
**Study type:**	Interventional Studies (Clinical Trials)
**Study Results:**	All Studies
**Intervention/treatment:**	resistance training OR strength training AND Exercise OR Aerobic Exercise
**Outcome Measure:**	Depressive Disorder, Major

https://clinicaltrials.gov/ct2/results?cond=Depressive+Disorder%2C+Major&term=randomized&type=Intr&rslt=&age_v=&gndr=&intr=resistance+training+OR+strength+training+AND+Exercise+OR+Aerobic+Exercise&titles=&outc=&spons=&lead=&id=&cntry=&state=&city=&dist=&locn=&rsub=&strd_s=&strd_e=&prcd_s=&prcd_e=&sfpd_s=&sfpd_e=&rfpd_s=&rfpd_e=&lupd_s=&lupd_e=&sort=

Table S1 *Studies excluded and reason for exclusion*

**Excluded studies with their respective reason**	
**Study**	**Reason for exclusion**
**Brüchle, 2021**<br>**Germany**	Interventions based on physical activity programs.
**Moncrieft, 2016**<br>**USA**	The study was conducted with diabetic individuals with depressive symptoms. They did not have a diagnosis of Major Depressive Disorder.
**Moraes, 2019**<br>**Brazil**	The study comparison was aerobic exercise and low-intensity resistance exercise.
**Pilu, 2007**<br>**Italy**	This was a case-control study. They also carried out interventions based on physical activity programs.
**Wa Chaua, 2020**<br>**Hong Kong**	Several of the participants in both the intervention and control groups were prescribed psychiatric medications.
**Knubben, 2007**<br>**Alemania**	The population did not have a diagnosis of major depression.
**Foley, 2008**<br>**New Zealand**	Included adults taking antidepressant medications.
**Haboush, 2006**<br>**USA**	This is a pilot study that conducted interventions with depressed older adults. However, they do not report whether they had a diagnosis of major depression.
**Salehi, 2016**<br>**Iran**	The comparator in this study is electroconvulsive therapy.
**Kerling, 2015**<br>**Germany**	Some of the individuals received regular therapy during the exercise interventions.
**Legrand, 2016**<br>**France**	During the interventions, the people were taking antidepressants.
**Abdelbasset, 2021**<br>**Saudi Arabia**	The population did not have a diagnosis of major depression.
**Zahra, 2016**<br>**Iran**	Drug interventions
**Akandere, 2011**<br>**Turkey**	The population did not have a diagnosis of major depression.
**Ansai, 2016**<br>**Brazil**	The population did not have a diagnosis of major depression.
**Moreira, 2005**<br>**Brazil**	The population did not have a diagnosis of major depression.
**Bernard, 2015**<br>**France**	Patients with Dysthymia
**Netz, 2017**<br>**Israel**	Literature review

**Zhang, 2022**<br>**China**	A cross-sectional
**Conradsson, 2010**<br>**Sweden**	The population did not have a diagnosis of major depression.
**Damush, 1999**<br>**USA**	The population did not have a diagnosis of major depression.
**Geliebter, 1997**<br>**USA**	The population did not have a diagnosis of major depression.
**Isaksen, 2016**<br>**Norway**	The population did not have a diagnosis of major depression.
**Jagger, 2015**<br>**UK**	The population did not have a diagnosis of major depression.
**Kekäläinen, 2018**<br>**Finland**	The population did not have a diagnosis of major depression.
**Kerse, 2010**<br>**Australia**	The population did not have a diagnosis of major depression.
**Legrand, 2007**<br>**France**	Aerobic exercise as a comparator
**Mendham, 2021**<br>**South Africa.**	The population did not have a diagnosis of major depression.
**Naumann, 2020**<br>**Germany**	Outpatients with moderate depression
**Neidig, 2003**<br>**USA**	The population did not have a diagnosis of major depression.
**Ng, 2017**<br>**Malaysia**	Cancer patients without a diagnosis of major depression.
**Norvell, 1993**<br>**USA**	The population did not have a diagnosis of major depression.
**Palmer, 1995**<br>**USA**	The population did not have a diagnosis of major depression.
**Payne, 2008**<br>**USA**	The population did not have a diagnosis of major depression.
**Perna, 2010**<br>**USA**	Cancer patients without a diagnosis of major depression.
**Puterman, 2021**<br>**Canada**	The population did not have a diagnosis of major depression.
**Tapps, 2013**<br>**USA**	Interventions based on physical activity programs.
**Vanková, 2014**<br>**Czech Republic**	The population did not have a diagnosis of major depression.
**Schuch, 2011**<br>**Brazil**	The study does not present the components of the FITTVP. Also, the intervention was accompanied by pharmacological therapy.
**Krogh, 2009**<br>**Denmark**	The population suffered from unipolar depression whose classification was based on the International Classification of Diseases, Tenth Revision (ICD-10).
**Singh, 2005**<br>**Australia**	Intervened with people diagnosed with depressive disorders (MDD, Dysthymia and minor depression).

**Hoffman, 2011 USA**	Participants were observed for one year after the interventions and reported antidepressant medication use.
**Sims, 2006 Australia**	The participants did not have a diagnosis of major depressive disorder. This study evaluated depressive symptomatology in older adults.
**Mota-Pereira, 2011 Portugal**	They compared aerobic exercise and medication with medication alone.
**Martinsen, 1985 Norway**	They compared aerobic exercise and medication with medication alone.
**Schuch, 2015 Brazil**	They compared aerobic exercise and medication with medication alone.
**Rueter, 1980 USA**	They compared aerobic exercise and therapy with therapy.
**Veale, 1992 UK**	Participants received the usual therapy (psychological and pharmacological) during the interventions.
**Oertel-Kno¨chel, 2014 Germany**	They included adults with MDD and schizophrenia who were taking medication for those illnesses.
**Babyak, 2000 USA**	The results of the study during the interventions were published by Blumenthal, 1999. This is the same intervention but published in different studies.

Fig. S1 *Risk of bias of individual studies*

Summary of risk of bias of studies

Intention-to-treat

Unique ID	Study ID	Experimental	Comparator	Outcome	Weight	D1	D2	D3	D4	D5	Overall
1	Blumenthal 1999	Aerobic Exercise	Medication	Depressive symptoms	NA	-	!	+	+	!	-
2	Blumenthal 2007	Aerobic Exercise	Medication	Depressive symptoms	NA	+	+	+	+	+	+
3	Dunn 2005	Aerobic Exercise	Placebo	Depressive symptoms	NA	+	!	+	-	!	-
4	Herman 2002	Aerobic Exercise	Medication	Depressive symptoms	NA	-	-	+	+	!	-
5	Khatri 2001	Aerobic Exercise	Medication	Depressive symptoms	NA	-	!	+	!	!	-
6	Krogh 2012	Aerobic Exercise	Stretching Exercise	Depressive symptoms	NA	+	!	+	+	+	!
7	Krogh 2014	Aerobic Exercise	Stretching Exercise	Depressive symptoms	NA	+	!	+	+	+	!
8	Sadeghi 2016	Aerobic Exercise	Cognitive Behavior	Depressive symptoms	NA	-	-	-	-	!	-
9	Singh 1996	Resistance Exercise	Health Education Program	Depressive symptoms	NA	!	!	+	-	!	-

+ Low risk
! Some concerns
- High risk

D1 Randomisation process
D2 Deviations from the intended interventions
D3 Missing outcome data
D4 Measurement of the outcome
D5 Selection of the reported result

Fig. S1 Risk of bias II graph: review authors' judgments about each risk of bias item presented as all included studies

Table S2 *Report of exercise interventions in physical exercise programs*

Report on the completeness of physical exercise interventions in the different modalities: resistance exercise and aerobic exercise			
**CERT Ítem**	**Total interventions (n= 13) n (%)**	**Resistance exercise (n= 1) n (%)**	**Aerobic exercise (n= 12) n (%)**
**1. What (materials)**	12 (92.30%)	1 (100%)	11 (91.66%)
**2. Who (Provider)**	5 (38.46%)	1 (100%)	4 (33.33%)
**3. Individual or group**	9 (69.23%)	0 (0%)	9 (75.00%)
**4. Supervised or unsupervised**	13 (100%)	1 (100%)	12 (100%)
**5. Adherence report**	8 (61.53%)	0 (0%)	8 (66.66%)
**6. Motivation strategies**	0 (0%)	0 (0%)	0 (0%)
**7a. Exercise progression**	5 (38.46%)	1 (100%)	4 (33.33%)
**7b. Program progression**	4 (30.76%)	1 (100%)	3 (25.00%)
**8. Replication of the exercise**	13 (100%)	1 (100%)	12 (100%)
**9. Home component**	2 (15.38%)	0 (0%)	2 (16.66%)
**10. Non-exercise components**	2 (15.38%)	1 (100%)	1 (8.33%)
**11.Adverse Event Reporting**	9 (69.23%)	1 (100%)	8 (66.66%)
**12. Environment**	5 (38.46%)	0 (0%)	5 (41.66%)
**13. Exercise description**	13 (100%)	1 (100%)	12 (100%)
**14a. Generic or personalized exercises**	13 (100%)	1 (100%)	12 (100%)

**14b. Adaptation of exercise to individuals**	13 (100%)	1 (100%)	12 (100%)
**15. Rules for the initial level**	13 (100%)	1 (100%)	12 (100%)
**16a. Adherence measurement**	8 (61.53%)	0 (0%)	8 (66.66%)
**16b. ¿Does the intervention match the planning?**	13 (100%)	1 (100%)	12 (100%)

CERT: The Consensus on Exercise Reporting Template tool.

Table S3 *Adverse events by degree of severity described for those in the aerobic exercise and controls*

**AEs Degrees**	**Aerobic exercise (includes supervised aerobic exercise and unsupervised home aerobic exercise) [25 adverse events, 375 participants] (Total de AEs[α] - exercise-related AEs)**	**Controls: treatment recommended by ACP (second-generation antidepressants or cognitive behavioral therapy), mind-body exercise, yoga, or tai-chi and no exercise interventions. [39 adverse events, 308 participants] (Total AEs[α] - AEs related to usual care)**
**1**	Grade 1 adverse events: (5 - 5)	Grade 1 adverse events: Null
	Muscle event (3-3)	
	Other painful manifestations-Chest pain (1-1)	
	Other painful manifestations-Muscle/joint pain (1-1)	
**2**	Grade 2 adverse events: (10 - 0)	Grade 2 adverse events: (14 - 1)
	Mood disturbances-Worsening MDD (6-0*)	Mood disturbances-Worsening MDD (6-1*)
	Mood disturbances-Antidepressants under follow-up (4-0*)	Mood disturbances-Antidepressants in follow-up (8-0*)
**3[b]**	Grade 3 adverse events: (9 - 1)	Grade 3 adverse events: (25 - 21)
	Unspecified medical reasons (3-0*)	Medication AE-Dizziness, drowsiness, agitation, diarrhea (19-19)
	Medical contraindication (1-1)	New Medical Condition (2-2)
	Mood disturbances-Psychiatric emergencies (3-0*)	Mood disorders-Psychiatric emergencies (2-0*)
	Mood disturbances-Admission to psychiatry (2-0*)	Mood disturbances-Admission to psychiatry (2-0*)
**4**	Grade 4 adverse events: (1 - 0*)	Grade 4 adverse events: Null
	Mood disturbances-Suicidal ideation (1-0*)	
**5**	Grade 5 adverse events: Null	Grade 5 adverse events: Null

AEs: Adverse Event; ACP: American College of Physicians; * =not related to the intervention The AEs were classified according to the Common Terminology Criteria: grade 1) mild or asymptomatic symptoms; grade 2) indicated moderate, minimal, local or non-invasive intervention and limitation of age-appropriate activities of daily living; grade 3) seriously or medically significant but not immediately life-threatening; grade 4) life-threatening consequences and indicated urgent intervention, or; Grade 5) Death

[α] Includes all AEs (both exercise-related and non-exercise-related)
[b] The AEs in those who were not reported to be grade 3.

Table S4 *Subgroup analysis*

Summary of results based on subgroups with one or more studies.								
**Outcomes**	**Subgroups**	**Studies**	**Sample**		**Std. Mean Difference IV, Random, 95% CI**	**Subgroup I²**	**P**	**Total I²**
			**IG**	**CG**				
**Depressive symptoms**								
	**Exercise modalities**	7	428	386	-0.27 [-0.58, 0.04]		P = 0.09	76%
	Aerobic Exercise	6	413	373	-0.26 [-0.59, 0.07]	78%	P = 0.12	76%
	Resistance Exercise	1	15	13	-0.43 [-1.18, 0.32]	Not applicable	P = 0.26	76%
	**Symptom assessment instrument**	7	428	386	-0.27 [-0.58, 0.04]		P = 0.09	76%
	Hamilton Depression Rating Scale	4	265	235	-0.46 [-0.94, 0.01]	83%	P = 0.06	76%
	Beck Depression Inventory	4	163	151	-0.02 [-0.31, 0.28]	41%	P = 0.92	76%
	**Body composition**	4	238	206	-0.55 [-1.06, -0.04]		P = 0.03	82%
	Overweight adults	2	71	72	-0.05 [-0.55, 0.45]	40%	P = 0.84	82%
	Obese adults	1	104	98	-0.08 [-0.36, 0.20]	0%	P = 0.57	82%
	Overweight and obese adults	1	63	36	-1.27 [-2.65, 0.12]	88%	P = 0.07	82%
	**Age comparison**	7	428	386	-0.27 [-0.58, 0.04]		P = 0.09	76%
	Under 50 years old	2	119	95	-0.94 [-2.02, 0.13]	90%	P = 0.09	76%
	Over 50 years old	5	309	291	-0.03 [-0.19, 0.14]	0%	P = 0.76	76%
	**Physical exercise frequency**	7	428	386	-0.27 [-0.58, 0.04]		P = 0.09	76%
	Three days per week	7	398	368	-0.15 [-	65%	P = 0.24	76%

					0.41, 0.10]			
	Five days per week	1	30	18	-1.13 [-4.22, 1.95]	94%	P = 0.47	76%
	**Exercise intensity**	7	428	386	-0.71 [-1.71, 0.28]		P = 0.16	76%
	Vo2 Max	1	56	59	0.12 [-0.24, 0.49]	Not applicable	P = 0.51	76%
	Reserve heart rate	4	294	278	-0.01 [-0.17, 0.16]	0%	P = 0.94	76%
	Low Intensity	1	31	18	-0.13 [-1.21, 0.94]	69%	P = 0.81	76%
	High Intensity	1	32	18	-2.45 [-3.23, -1.67]	0%	P < 0.00001)	76%
	Repetition maximum	1	15	13	-0.43 [-1.18, 0.32]	Not applicable	P = 0.23	76%
	**Total working time per week**	7	428	386	-0.27 [-0.58, 0.04]		P = 0.09	76%
	90 minutes	6	383	355	-0.13 [-0.40, 0.13]	67%	P = 0.32	76%
	150 minutes	1	30	18	-1.13 [-4.22, 1.95]	94%	P = 0.47	76%
	180 minutes	1	15	13	-0.43 [-1.18, 0.32]	Not applicable	P = 0.26	76%
	**Duration of interventions**	7	428	386	-0.27 [-0.58, 0.04]		P = 0.09	76%
	8 - 12 weeks	3	134	108	-0.84 [-1.69, 0.02]	87%	P = 0.06	76%
	16 weeks	4	294	278	-0.01 [-0.17, 0.16]	0%	P = 0.94	76%
	**Control conditions**	7	428	386	-0.27 [-0.58, 0.04]		P = 0.09	76%
	Medication	4	294	278	-0.01 [-0.17, 0.16]	0%	P = 0.94	76%
	Flexibility	2	119	95	-0.94 [-2.02, 0.13]	90%	P=0.09	76%
	Non-Exercise	1	15	13	-0.43 [-1.18, 0.32]	Not applicable	P = 0.26	76%
	IG: Intervention Group; CG: Control Group; IC: confidence interval; I²: heterogeneity test; P: statistical							

	significance.

Fig. S2 *Quality of life*

	Physical Exercise			Control				Std. Mean Difference
Study or Subgroup	Mean	SD	Total	Mean	SD	Total	Weight	IV, Random, 95% CI
Krogh, 2012	41.3	24	56	42.8	25.5	59	78.3%	-0.06 [-0.43, 0.31]
Singh, 1996	63.9	26.5	17	63.5	23.9	15	21.7%	0.02 [-0.68, 0.71]
**Total (95% CI)**			**73**			**74**	**100.0%**	**-0.04 [-0.37, 0.28]**

Heterogeneity: Tau² = 0.00; Chi² = 0.04, df = 1 (P = 0.85); I² = 0%
Test for overall effect: Z = 0.26 (P = 0.79)

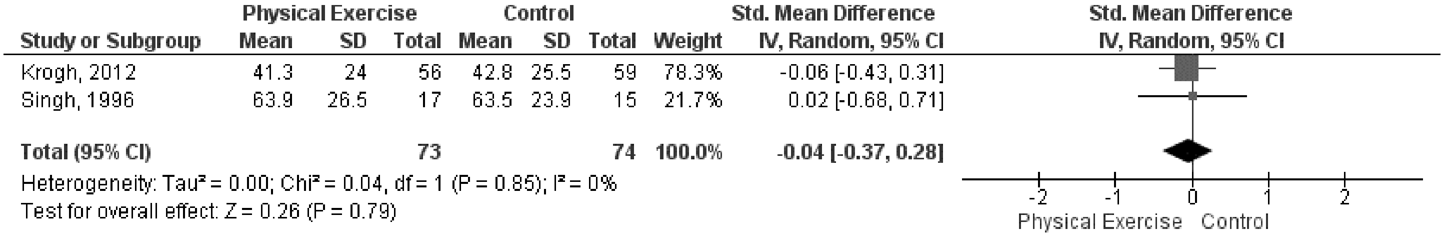

Fig. S2 Analysis of the effect of physical exercise programs on Quality of life in adults with major depressive disorder compared with control. I^2: heterogeneity, *p* value <0,05, Standardized mean difference, Random effects model

Table S5 *Summary of findings*

Physical exercise compared to second-generation antidepressants, BME or no exercise interventions in adults with major depressive disorder who do not receive treatment (second-generation antidepressants or cognitive behavioral therapy)

Patient or population: adults with major depressive disorder who do not receive treatment (second-generation antidepressants or cognitive behavioral therapy)
Intervention: physical exercise
Comparison: second-generation antidepressants, BME or no exercise interventions

Outcomes	**Anticipated absolute effects*** (95% CI)		Relative effect (95% CI)	№ of participants (studies)	Certainty of the evidence (GRADE)	Comments
	**Risk second-generation antidepressants, BME or no exercise interventions**	**Risk with physical exercise**				
Depressive symptoms assessed with: Questionnaires (HAM-D, BDI, BDI-II) follow-up: range 8 weeks to 16 weeks	The mean depressive symptoms ranged from **6.29 a 23.3** score	SMD **0.27 score fewer (0.58 fewer to 0.04 more)**	-	814 (7 RCTs)	⊕◯◯◯ Very low[a,b,c,d]	The physical exercise may reduce little to no effect on depressive symptoms, but the evidence is very uncertain
Adverse events assessed with: Common Terminology Criteria for adverse events	105 per 1.000	**0 per 1.000 (0 to 0)**	not estimable	812 (7 RCTs)	⊕⊕◯◯ Low[a,d,e]	The evidence suggests that physical exercise does not increase adverse events
Quality of life assessed with: Questionaries (SF-36, WHQ-5)	The mean quality of life ranged from **42.8 a 63.5** score	SMD **1.14 score fewer (9.28 fewer to 7 more)**	-	143 (2 RCTs)	⊕◯◯◯ Very low[c,d,f,g]	The physical exercise may have little to no effect on quality of life, but the evidence is very uncertain
Mortality - not reported	-	-	-	-	-	

***The risk in the intervention group** (and its 95% confidence interval) is based on the assumed risk in the comparison group and the **relative effect** of the intervention (and its 95% CI). **CI:** confidence interval; **MD:** mean difference

GRADE Working Group grades of evidence.
High certainty: we are very confident that the true effect lies close to that of the estimate of the effect.
Moderate certainty: we are moderately confident in the effect estimate: the true effect is likely to be close to the estimate of the effect, but there is a possibility that it is substantially different.
Low certainty: our confidence in the effect estimate is limited: the true effect may be substantially different from the estimate of the effect.
Very low certainty: we have very little confidence in the effect estimate: the true effect is likely to be substantially different from the estimate of effect.
